# β3-Adrenoreceptor Stimulation Protects against Myocardial Infarction Injury *via* eNOS and nNOS Activation

**DOI:** 10.1371/journal.pone.0098713

**Published:** 2014-06-09

**Authors:** Xiaolin Niu, Lianyou Zhao, Xue Li, Yusheng Xue, Bin Wang, Zongqiang Lv, Jianghong Chen, Dongdong Sun, Qiangsun Zheng

**Affiliations:** 1 Department of Cardiology, Tangdu Hospital, the Fourth Military Medical University, Xi'an, Shaanxi, China; 2 Department of Cardiology, Xijing Hospital, the Fourth Military Medical University, Xi'an, Shaanxi, China; 3 Cadet Brigade of Fourth Military Medical University, Xi'an, Shaanxi, China; Goethe University, Germany

## Abstract

β3-adrenergic receptor (AR) and the downstream signaling, nitric oxide synthase (NOS) isoforms, have been emerged as novel modulators of heart function and even potential therapeutic targets for cardiovascular diseases. However, it is not known whether β3-AR plays cardioprotective effects against myocardial infarction (MI) injury. Therefore, the present study was designed to determine the effects of β3-AR on MI injury and to elucidate the underlying mechanism. MI model was constructed by left anterior descending (LAD) artery ligation. Animals were administrated with β3-AR agonist BRL37344 (BRL) or β3-AR inhibitor SR59230A (SR) respectively at 0.1 mg/kg/hour one day after MI operation. The scar area, cardiac function and the apoptosis of myocardial were assessed by Masson's trichrome stain, echocardiography and TUNEL assay respectively. Western blot analysis was performed to elucidate the expressions of target proteins. β3-AR activation with BRL administration significantly attenuated fibrosis and decreased scar area after MI. Moreover, BRL also preserved heart function, and reduced the apoptosis of cardiomyocyte induced by MI. Furthermore, BRL treatment altered the phosphorylation status of endothelial NOS (eNOS) and increased the expression of neuronal NOS (nNOS). These results suggested that β3-AR stimulation has a substantial effect on recovery of heart function. In addition, the activations of both eNOS and nNOS may be associated with the cardiac protective effects of β3-AR.

## Introduction

Despite a wide range of therapeutic approaches, myocardial infarction (MI) continues to be one of the leading cause of death worldwide [1], [2], [3]. Therefore, finding an effective therapeutic target is important to reduce MI injury. In the heart, β-adrenoceptors (β-AR) are primary regulators for cardiac performance in response to stress [4]. The effects of β1-AR and β2-AR are well established which play positive chronotropic and inotropic effects [4], [5].β3-AR, mediating lipolysis and thermogenesis effects in adipocytes, has been considered as a potential antiobesity and antidiabetic treatment [6], [7]. In contrast to the well-characterized β1/β2-AR, β3-AR can be also activated by catecholamines but at higher concentrations [8].

Recently, accumulating evidence demonstrated that β3-AR presents in the endothelium and myocardium. Moreover, β3-AR stimulation regulates specific effects in the cardiovascular system, including positive chronotropic effect, vasodilation effect and even affecting the electrical properties of the heart [9], [10], [11]. Therefore, β3-AR has been emerged as a potential therapeutic target for cardiovascular diseases. Furthermore, it is well established that β3-AR is upregulated in failing hearts and diabetic hearts [12]. Belge et al. showed that β3-AR over-expression was associated with attenuated left ventricular (LV) hypertrophy induced by chronic isoprenaline treatment. By contrast, we have previously demonstrated that β3-AR^−/−^ mice exhibited exacerbated pathological remodeling and impaired cardiac functional [10]. These results suggested that β3-AR may play a protective role in pathological remodeling and in the development of heart failure.

There is accumulating evidence suggesting that β3-AR plays an important role in the modulation of cardiovascular function in heart failure that. Moreover, these effects may be associated with nitric oxide (NO) release via nitric oxide synthase (NOS) activation [13]. Previous studies have shown that β3-AR stimulation increases NO release [14]. Furthermore, the cardiac effects of β3-AR agonist, BRL 37344 (BRL), were abolished by NOS inhibitor [15], indicating that the cardiac-protective effects of β3-AR are regulated by NOS-NO signaling. Several studies have suggested that endothelial NOS (eNOS) is solely responsible for β3-AR induced cardiac signaling. By contrary, nNOS was up-regulated in failing hearts after MI [16], indicating that nNOS-NO pathway may be also associated with β3-AR induced cardiac regulation. However, the potential role of nNOS still remains unclear.

MI, inducing permanent loss of cardiomyocyte mass and pathological left ventriclar remodeling, is the major cause of heart failure [3]. Moreover, MI is also associated with sustained over-activation of sympathetic nervous system, which results in an increased β3-AR stimulus (ie, catecholamines). However, it is not known whether β3-AR plays cardiac-protective effects against MI injury. Therefore, we designed the present study to explore the potential role of β3-AR in cardiac physiology and pathobiology during MI and also to elucidate the underline mechanism.

## Methods

### Animals

One hundred and twenty adult male C57BL6/J mice (weighing 20 to 25 g, 8–10 weeks) were purchased from the animal centre in the Fourth Military Medical University. Mice were housed in a temperature-controlled animal facility with a 12-hour light/dark cycle, with tap water and rodent chow provided ad libitum. Mice were randomly allocated into the following groups with n = 30 each: (1) sham group (Sham); (2) MI group (MI); (3) MI + BRL37344 group (MI+BRL); (4) MI + SR59230A group (MI+SR). The experiments were performed in adherence with the National Institutes of Health Guidelines on the Use of Laboratory Animals and were approved by the Fourth Military Medical University Committee on Animal Care.

### Construction of myocardial infarction model

Myocardial infarction (MI) was constructed by left anterior descending (LAD) artery ligation as described previously [17]. In brief, a left thoracotomy was performed between ribs 2 and 4 under anesthesia with 2% isoflurane. LAD was permanently ligated with a 6–0 suture. The ligation was deemed successful by characteristic ECG changes. Moreover, sham operated control mice underwent the same surgical procedures except that the suture under the left coronary artery was not tied.

Mice in MI+BRL and MI+SR groups were administrated with BRL37344 or SR59230A respectively at 0.1 mg/kg/hour via osmotic mini-pumps (Alzet Inc, Cupertino, CA) one day after MI operation.

### Histological evaluation of fibrosis

Mice were sacrificed for histological assay 4 weeks after MI operation. Hearts were fixed in 4% paraformaldehyde and embedded in paraffin. Serial sections were prepared at 5 µm thickness, and Masson's trichrome stain was performed to detect fibrosis in cardiac muscle. Ten anterolateral sections from each heart were evaluated in their entirety and quantified. Computerized morphometry was used to calculate the scar extent as the ratio of scar and total left ventricular area using Imaging Pro Plus software.

### Echocardiographic measurements of cardiac function

Echocardiography studies were conducted at 2 days, 7days and weekly until sacrificed at 4 weeks after infarction as previously described [18]. Mice were anesthetized (2% isoflurane and oxygen) and put in a supine position. Both two-dimensional and M-mode images were recorded using a 30-MHz transducer on a Vevo 2100 ultrasound system (VisualSonics, CA). The left ventricular end-systolic diameter (LVESd) and left ventricular end-diastolic diameter (LVEDd) were measured to calculate left ventricular ejection fraction (LVEF) and fractional shortening (FS). Echocardiography was evaluated in a blinded manner.

### Determination of myocardial apoptosis

Myocardial apoptosis was determined by terminal deoxynucleotidyl transferase-mediated dUTP-biotin nick end labeling (TUNEL) assay and caspase-3 activity assay as previously described [19]. In brief, serial sections of heart tissue were stained with fluorescein-dUTP (In Situ Cell Death Detection Kit; Roche Diagnostics) for apoptotic cell and stained with 4′,6-diamidino-2-phenylindole (DAPI) (Sigma) for all cell nuclei. The percentage of apoptotic cells was termed as the apoptotic index.

Caspase-3 activity was determined with a fluorometric caspase-3 assay plate (Clontech, Mountain View, Calif) according to the manufacturer's instructions [19]. In brief, assay plates were incubated at 37°C for 1 h, and the substrate cleavage was measured fluorometrically with a spectrophotometer set at 360 nm excitation and 460 nm emission.

### Western blot assay

Western blotting was performed following standard protocol as described previously [20]. Protein was isolated from homogenized LV tissue using cell lysis buffer (Cell Signaling Technology, Danvers, MA). Equal amounts of protein sample (50 µg) were separated by electrophoresis on 12% SDS-PAGE gels in a Tris/HCl buffer system, and sequentially electrophoretically transferred to nitrocellulose (NC) membranes. After blocking with Tris-Buffered Saline Tween-20 (TBST) containing 5% milk (TBST: milk), NC membranes were subjected to immunoblotting with appropriate primary antibodies over night at 4°C, followed by incubation with appropriate secondary antibody conjugated with horseradish peroxidase at 37°C for 60 min. The following primary antibodies were used: eNOS (1∶1000,Cell Signaling Technology); Phospho-eNOS Ser1177 (p-eNOS ^Ser1177^) (1∶1000, Cell Signaling Technology); Phospho-eNOS Thr495 (p-eNOS ^Thr495^) (1∶1000, Cell Signaling Technology); Phospho-eNOS Ser114 (p-eNOS ^Ser114^) (1∶1000, Cell Signaling Technology); Phospho-eNOS Ser633 (p-eNOS ^Ser633^) (1∶500, Abcam); iNOS (1∶1000, Cell Signaling Technology); Phospho-nNOS Ser1417 (p-nNOS ^Ser1417^) (1∶500, Abcam); Phospho-nNOS Ser847 (p-nNOS ^Ser847^) (1∶500, Abcam); nNOS (1∶1000, Cell Signaling Technology); β-actin (1: 5000, Abcam); β1-adrenergic receptor (1∶500, Abcam); β2-adrenergic receptor (1: 500, Abcam); β3-adrenergic receptor (1: 500, Abcam). Blots bands were visualized with an enhanced chemiluminescene system (Amersham Bioscience, Buchinghamshire, UK), and densitometric analysis of Western blots was carried out using VisionWorks LS, version 6.7.1.

### RNA extraction and Reverse Transcription - Polymerase Chain Reaction (RT-PCR) analysis

The mRNA expressions of nNOS were assessed by reverse transcription PCR (RT-PCR) assay. (See Methods S1)

### Statistical analysis

All data are presented as mean ± SEM. Statistics were calculated using Prism 5.0 (GraphPad Software Inc, San Diego, CA, USA). Statistical comparisons for different groups were performed using one-way ANOVA followed by Student's paired, two-tailed t-test for two groups' comparison. *P* values <0.05 were considered statistically significant.

## Results

### β3-AR stimulation reduced fibrosis after MI

**Figure 1:**
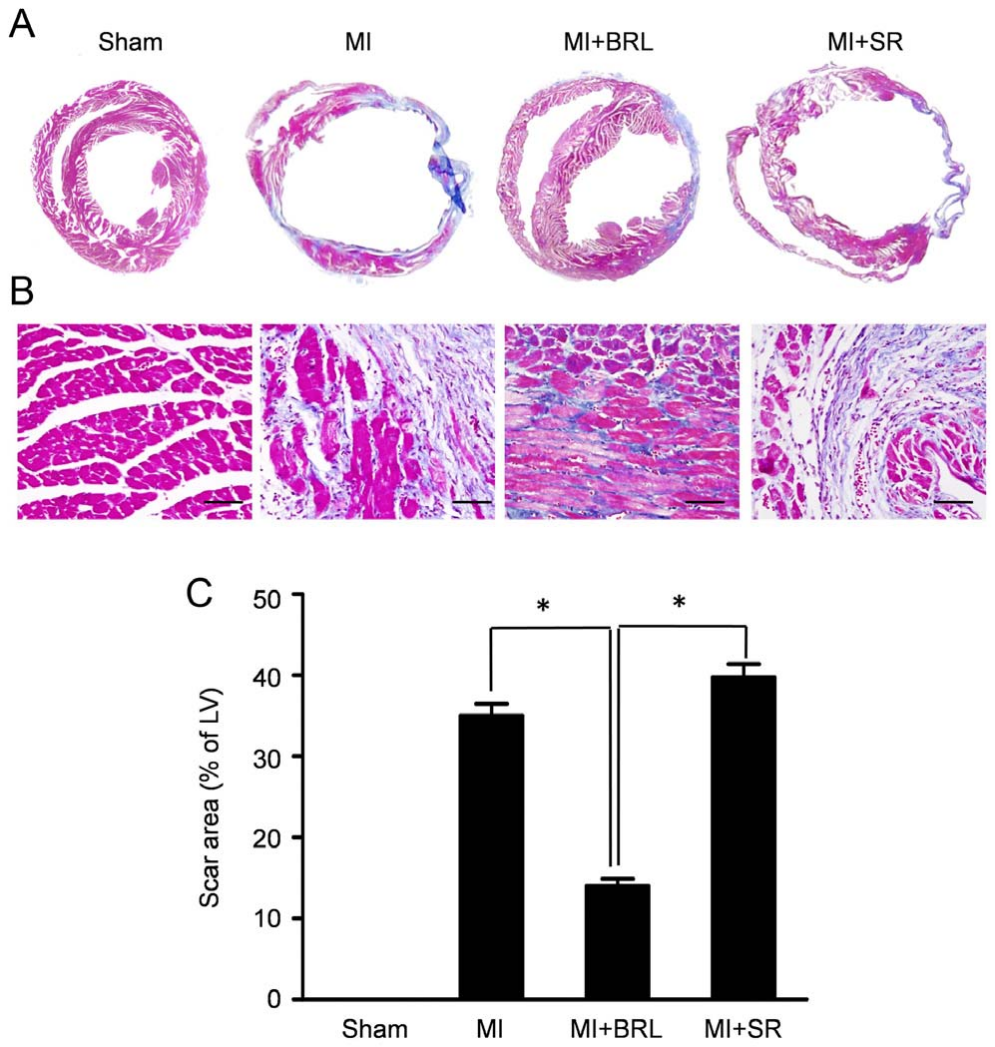
Effect of BRL and SR on left ventricular fibrosis induced by MI. (A) Representative Masson's trichrome staining revealed left ventricular fibrosis 4 weeks after MI (magnification: 4x). (B)Histological examination of fibrosis by Masson's trichrome staining in the border zone. Red indicates viable myocardium; blue indicates fibrosis due to infarction damage. Scale bar represents 50 µm. (C) Quantitative analysis of the scar area (*p<0.05).

To reveal the effects of β3-AR on the extent of fibrosis after MI, we performed Masson's trichrome staining. As illustrated in Figures 1A and 1B, severe fibrosis was observed in the hearts of MI group (35.21± 1.47%). Conversely, mice receiving β3-AR specific agonist, BRL-37344, displayed a 60% reduction in infarct area when compared to the MI group (14.03±0.91% *vs*. 35.21±1.47% in MI group, *P*<0.05). Moreover, β3-AR specific inhibitor SR59230A increased the scar area, although without significance (39.75±1.65% *vs*. 35.21±1.47% in MI group, *P*>0.05) (Figure. 1C).

### β3-AR specific agonist BRL-37344 preserved heart functional recovery after MI

**Figure 2:**
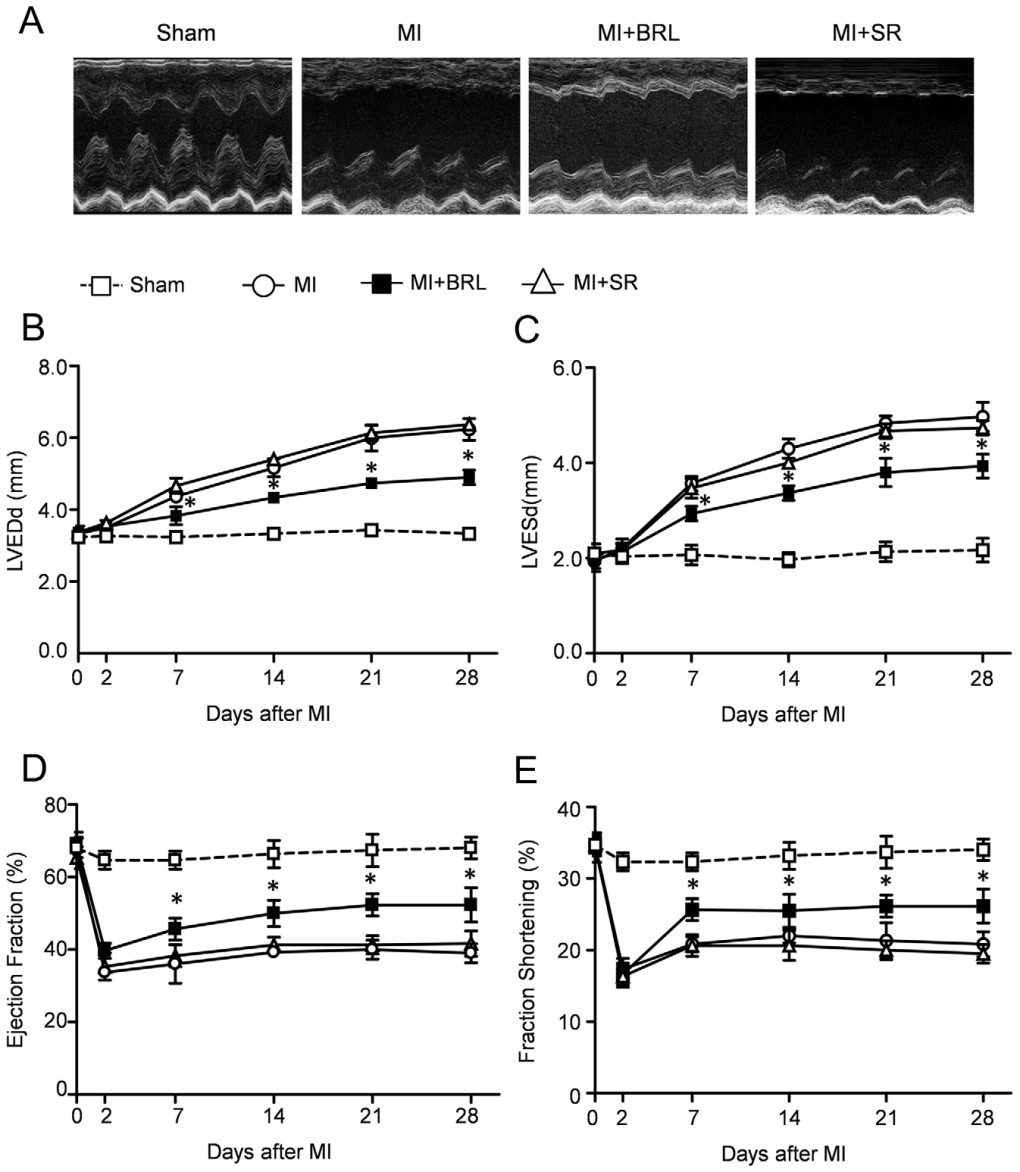
Effect of BRL and SR on LV dilation and LV systolic function after MI. (A) Representative M-mode echocardiography images were taken at the level of the papillary muscle where left ventricular diameters can be measured. Quantification of left ventricular end diastolic diameter (LVEDd) (B), end systolic diameter (LVESd) (C), left ventricular ejection fraction (EF) (D) and fractional shortening (FS) (E) 4 weeks after MI. (*p<0.05 *vs*. MI.)

We performed echocardiogram to evaluate the heart function after MI in all groups. Representative M-mode echocardiographic illustrations at 4 weeks after MI (Fig. 2A) revealed systolic dysfunction in MI and MI+SR group. Conversely, the LV anterior wall motion was obviously improved in MI+BRL group compared with MI group, indicating that BRL-37344 treatment manifested a trend towards improvement of cardiac function after MI. Serial echocardiographic analysis revealed that the baseline parameters were similar in all groups. In addition, the LVEDd and LVESd in MI mice were increased compared with Sham. Similarly, the LV dimensions in MI+SR group were also significantly increased. However, BRL-37344 treatment significantly decreased LVEDd and LVESd compared with MI group and MI+SR group (Figure 2B and 2C). Furthermore, the LVEF and FS were significantly enhanced in MI+BRL group compared with MI group and MI+SR group, suggesting that BRL-37344 treatment also preserved cardiac systolic function after MI (Figure 2D and 2E). No significant difference of LVEF and FS was observed in the MI mice that received vehicle or SR.

### β3-AR stimulation inhibited cardiomyocyte apoptosis after MI

**Figure 3:**
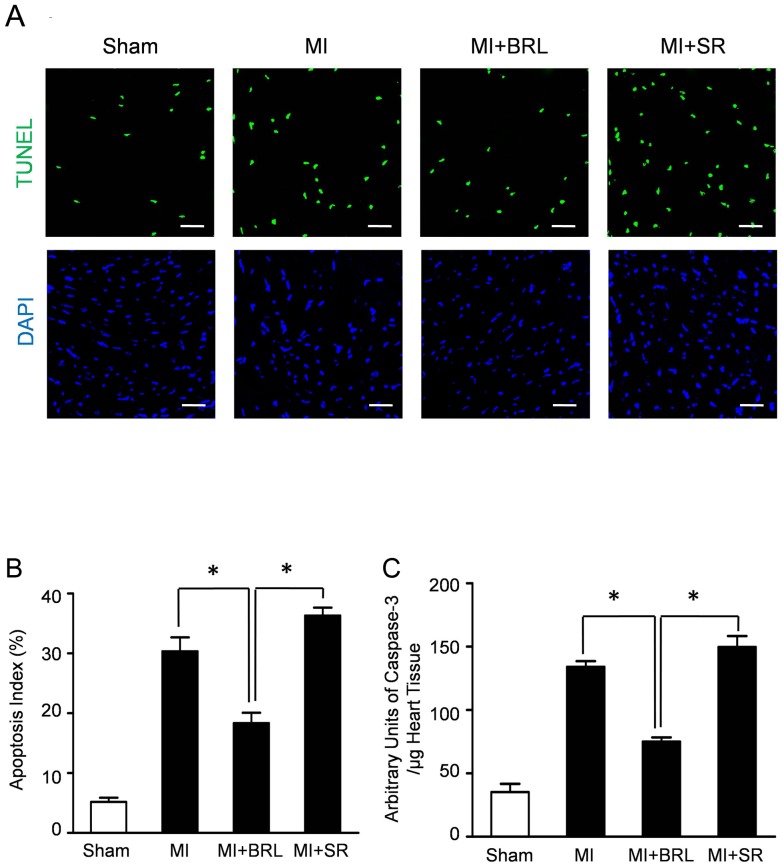
β3-AR stimulation decreased cardiomyocyte apoptosis. (A) Representative photographs of TUNEL-stained heart sections from sham group, MI group, MI+BRL group and MI+SR group. Apoptotic nuclei were identified as TUNEL positive (green fluorescent). Myocardium was stained using a monoclonal antibody against Troponin I (red fluorescent) and total nuclei was stained by DAPI (blue fluorescent). Scale bar represents 50 µm. (B) Apoptotic cells were quantified by apoptotic index (AI) which was termed as the percentage of apoptotic cells. (C) BRL administration also significantly decreased caspase-3 activity compared with MI group and MI+SR group (*p<0.05).

Representative immunofluorescence photomicrograph in figure 3A revealed that the apoptotic cardiomyocytes were more frequently observed in MI group and MI+SR group compared with that in the MI+BRL group. Quantitative analyses demonstrated that the apoptosis index in MI+BRL group was 18.33±1.76%, significantly less than that in MI group (30.33±2.33%, *P*<0.05) and that in MI+SR group (36.43±1.37%, *P*<0.05). However, no significance of apoptosis index was observed between MI group and MI+SR group (Figure. 3B).

Concurrently, BRL-37344 treatment significantly decreased caspase-3 enzymatic activity compared with MI group (75.28±3.46 vs 134.02±4.63, *P*<0.05) and MI+SR group (75.28±3.46 vs 149.78±8.67, *P*<0.05) (Figure 3C).

### Cardiac β3-AR expressions after MI

**Figure 4:**
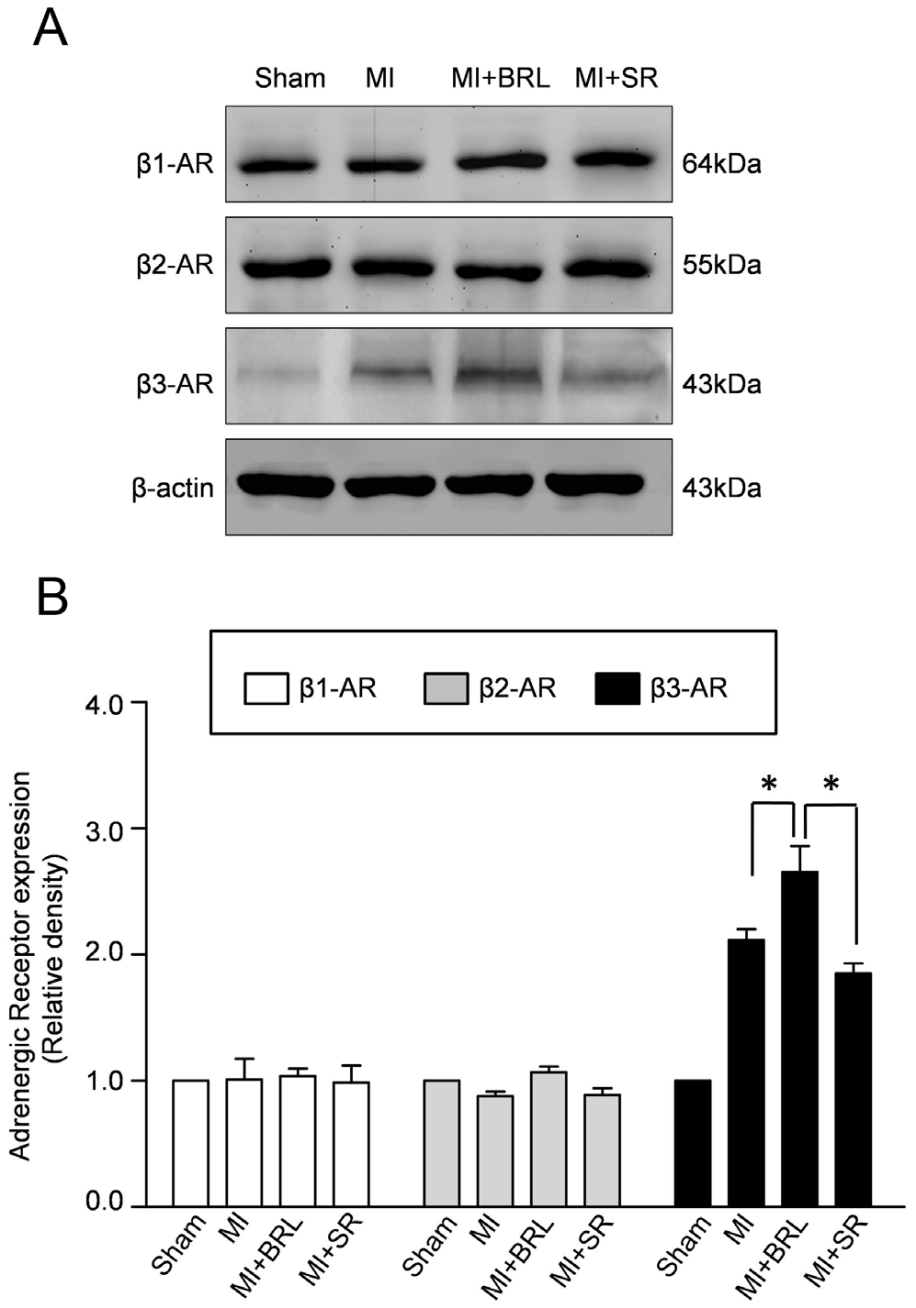
Effect of BRL and SR on the expressions of β-AR subtypes. (A) Representative immunoblots of β1-AR, β2-AR and β3-AR in sham, MI, MI+BRL and MI+SR groups. (B) Semiquantitative analysis of the expressions of β1-AR, β2-AR and β3-AR (*p<0.05).

Western blotting assay was performed to investigate the cardiac expressions of β-adrenoreceptors, including β1/β2-AR and β3-AR. As representative bloting results and semiquantitative analyses shown in Figure 4A,B, the expression of β3-AR was significantly increased in MI group, compared with sham. Moreover, the cardiac expression of β3-AR in MI+SR group was decreased than that in MI group without significant difference. Conversely, BRL-37344 treatment increased β3-AR expression. Furthermore, no changes in the expressions of β1-AR and β2-AR were noted in all groups.

### β3-AR modulated the activation of eNOS and regulated nNOS protein expression

**Figure 5:**
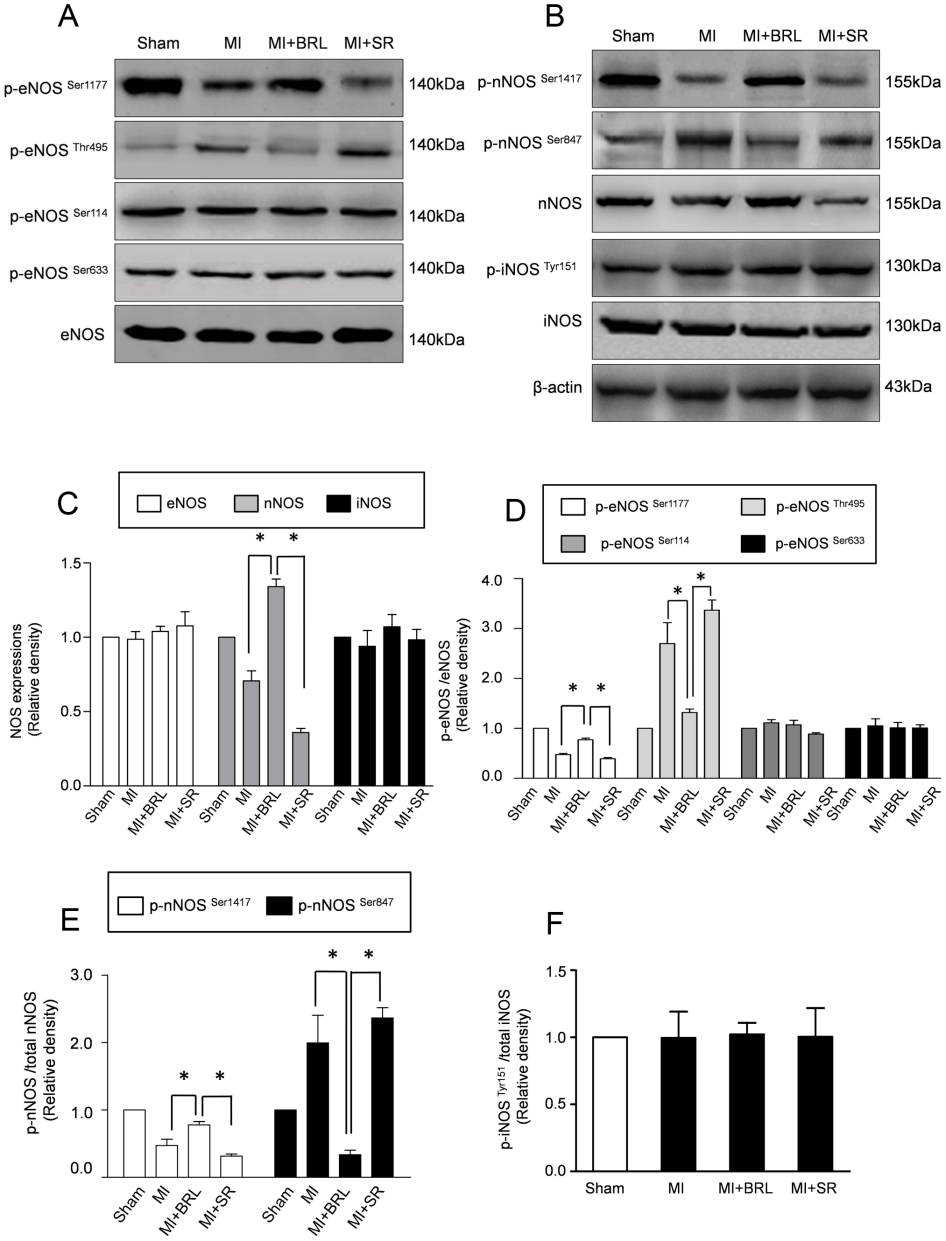
β3-AR stimulation altered the phosphorylation status of eNOS and increased the expression of nNOS. (A) Representative immunoblots of *p*-eNOS (Ser1177/Thr495/Ser114/Ser633) and total eNOS in sham, MI, MI+BRL and MI+SR groups. (B) Representative immunoblots of *p*-nNOS (Ser1417/Ser847), total nNOS, *p*-iNOS, iNOS and β-actin in all groups. Semiquantitative analysis of the expressions of eNOS, nNOS, iNOS(C), *p*-eNOS (Ser1177/Thr495/Ser114/Ser633) (D), *p*-nNOS(Ser1417/Ser847) (E) and *p*-iNOS (Tyr151) (F) (*p<0.05).

Previous studies suggested that β3-AR stimulation results in NO production via three NOS isoforms (i.e. eNOS, nNOS, and iNOS). Therefore, we evaluated the expressions of NOS isoforms after MI, as well as the role they played in the cardioprotective effects of β3-AR. First, we examined the eNOS expression and activation which is generally modulated by 4 phosphorylation sites: eNOS^Ser1177^, eNOS^Ser114^, eNOS^Ser633^ and eNOS^Thr495^. As representative bloting results and semiquantitative analyses shown in Figure 5A, D, total eNOS, phosphorylated eNOS^Ser114^ and phosphorylated eNOS^Ser633^ were unchanged in all groups. Moreover, phosphorylation of eNOS^Ser1177^, which indicates eNOS activation, significantly decreased in MI group and MI+SR group, whereas the expressions of phosph-eNOS^Thr495^ increased in MI group and MI+SR group. Furthermore, BRL-37344 treatment increased the expression of phosph-eNOS^Ser1177^, and decreased the level of phosph-eNOS ^Thr495^.

To furtherly investigate the role of β3-AR on other NOS isoforms, we performed the reverse transcription PCR (RT-PCR) to evaluate the mRNA expression of nNOS. The representative results in Figure S1 revealed that the mRNA expression of nNOS was significantly increased in MI +BRL group, compared with MI group and MI+SR group. These results indicated that the modulation of β3-AR on nNOS may be in a transcription way. Moreover, we examined the protein expressions of nNOS, and iNOS. As representative bloting (Figure 5B) and semiquantitative analyses in figure 5C, increased expressions of nNOS were observed in MI group and MI+SR group compared with sham group. Meanwhile, the expressions of phospho-nNOS^Ser1417^ were also increased in MI group and MI+SR group. However, MI increased the expression of phospho-nNOS^Ser847^. Furthermore, BRL-37344 treatment resulted in a 2-fold increase in nNOS protein expression. Moreover, BRL-37344 treatment increased the expression of phospho-nNOS^Ser1417^ and decreased the phospho-nNOS^Ser847^ expression compared with MI group (Figure 5B and E). In contrary, no difference of iNOS and phospho-iNOS expressions was observed in all groups (Figure 5B and F).

## Discussion

In the present study, we found that β3-AR activation decreased the apoptosis of cardiomyocytes and also inhibited fibrosis, which contributed to the enhanced cardiac function after MI. Moreover, our data revealed a novel mechanism by which eNOS and nNOS pathways were related with the protective effect of β3-AR.

In the heart, 3 subtypes of β-ARs, belonging to the G protein–coupled receptor superfamily, potentially play an essential role in modulating cardiac function [8]. The effects of β1- and β2-ARs are well established in mammals, including positive chronotropic and inotropic effects. Although the precise roles of β3-ARs remain uncertain, accumulating evidence suggests that β3-AR may play a negative inotropic effect which antagonizes the effects of β1/2-ARs [21]. Moreover, previous studies suggested that β3-AR is activated to act as a protective mechanism during sympathetic overstimulation conditions at higher concentrations of catecholamines than those required for β1/2-ARs [8], [14], [22]. Furthermore, our previous study demonstrated that β3^–/–^ mice exhibited exacerbated hypertrophy and cardiac systolic dysfunction induced by pressure-overload [10], [16]. Similarly, β1-blocker nebivolol, also a select β3-AR agonist, reduced infarct size in WT mice subjected to myocardial ischemia and reperfusion (MI/R) injury [23]. Moreover, the protective effects of nebivolol were completely abolished in β3^–/–^ mice [24]. These data suggested that β3-AR may play a significant role in regulating cardiac function and remodeling, the detailed effect of β3-AR on MI injury remains unclear. In the present study, we administered the β3-AR specific agonist BRL to MI mice and found that β3-AR stimulation prevented cardiac dysfunction with decreased fibrosis and myocardial apoptosis induced by MI. These results suggested that β3-AR agonist may constitute a potential and novel approach in treating pathological remodeling and heart failure after MI.

Although the mechanism of the protective effects of β3-AR is not clarified, previous studies have suggested that β3-AR stimulation increases eNOS activity and NO release [24], [25]. Furthermore, eNOS deficiency exacerbates myocardial I/R injury which is abolished by eNOS overexpression [26], [27], indicating the role of eNOS in β3-AR-induced cardiac protection. However, three NOS isoforms (i.e. eNOS, nNOS, and iNOS) are involved in NO release, which is involved in the regulation of myocardial function [28]. Previous studies also indicated that the nNOS expression and nNOS-derived NO levels are both increased in failing hearts [29], [30]. Meanwhile, some studies have demonstrated that β3-AR modulates NO signaling through nNOS or iNOS [16], [24]. There remains great controversy over which NOS isoform is the chief player in β3-AR signaling.

The activity of eNOS is generally modulated by either translocation or phosphorylation. We previously observed the β3-AR induced eNOS translocation only in the right atrium [16]. The activity of eNOS can be modulate by phosphorylation sites,including serine residue 1177 (phosph-eNOS^Ser1177^),threonine residue 497 (phosph-eNOS^Thr495^), serine residue 633(phosph-eNOS^Ser633^) and serine residue 114(phosph-eNOS^Ser114^) [31]. Phosphorylation at Ser1177 activates eNOS, whereas phosphorylations at Ser114 and Thr 497 inhibit the eNOS activity [32], [33]. In the present study, we observed that MI injury decreased the expression of phosph-eNOS^Ser1177^ and increased the expression of phosph-eNOS^Thr495^. However, β3-AR stimulation with BRL significantly increased phosph-eNOS^Ser1177^ and decreased phosph-eNOS^Thr495^. Interestingly, the phosphorylations of eNOS at Ser114 and Ser633 were unaltered after MI with or without BRL treatment. The alterations in the phosphorylation status of eNOS suggested that MI inhibited the eNOS activation which was abolished by β3-AR stimulation. Moreover, the expression of iNOS was unchanged by BRL treatment, which was consistent with our previous results [16].

Emerging evidence demonstrated that nNOS-derived NO production plays a substantial role in regulating myocardial contraction [30], [34], [35]. Moreover, the expression of nNOS was up-regulated in failing hearts after MI [34]. Furthermore, nNOS ^−/−^ mice exhibit more severe remodeling and functional deterioration induced by MI [30]. These results indicate that nNOS may be also involved in the protective response to MI injury. Previous studies suggested that phosphorylation at Ser 1417 activates nNOS and enhances the production of NO, followed by an inactivating phosphorylation at Ser847 [36], [37]. Our present results demonstrated that BRL-37344 treatment increased the expressions of nNOS and the phospho-nNOS, compared with MI group. However, β3-AR stimulation had no effect on the expressions of iNOS and phospho-iNOS. Taken together, these results provided interesting evidence that β3-AR stimulation with BRL acts as a dual activator of eNOS and nNOS. Consistently, John W. Calvert [38] observed that administrations of select β3-AR agonist nebivolol or CL 316243 increased the expression of nNOS and reduced myocardial I/R injury. By contrast, previous studies suggested that eNOS was the sole cardiac source of NO after β3-AR activation [25], [39]. Therefore, additional experiments are necessary to confirm the role of nNOS in MI injury and in the protection of β3-AR.

Although our study bears some clinical relevance, there are many limitations. First, the detailed physiologic and pathologic functions of β3-AR have not been extensively characterized. Moreover, there are still intense controversies about which NOS isoform is the chief player in β3-AR signaling. Therefore, further studies defining the exact mechanism are needed. In conclusion, we provided evidence that the administration of β3-AR specific agonism has a substantial effect on recovery of heart function. In addition, the activations of both eNOS and nNOS may be associated with the protective effects of β3-AR. These data collectively indicate that β3-AR stimulation, as a therapeutic target, may have clinical potential for myocardial infarction.

## Supporting Information

Methods S1 **RNA extraction and Reverse Transcription - Polymerase Chain Reaction (RT-PCR) analysis.** Total RNA was extracted using TRIZOL reagent (Invitrogen) in accordance with the manufacturer's protocol. After reverse-transcribed, cDNA samples were subjected to polymerase chain reaction (PCR) amplification. The following primers were used: The primer sequence for nNOS: forward 5′-GGC ACT GGC ATC GCA CCC TT-3′, reverse 5′-CTT TGG CCT GTC CGG TTC CC-3′. The β-actin housekeeping gene was used as an endogenous internal control. The primers for β-actin: forward 5′-AAC CGC GAG AAG ATG ACC CAG ATC ATG TTT-3′; reverse, 5′-AGC AGC CGT GGC CAT CTC TTG CTC GAA GTC-3′. The PCR products were fractionated by 1.2% agarose gel electrophoresis and visualized under UV illumination after staining with ethidium bromide.
(DOC)

Figure S1 **RT-PCR analysis of nNOS mRNA expression.** A: PCR-based detection of nNOS in sham group,MI Group, MI+SR group and MI+BRL group. B: Semiquantitative analysis of the mRNA expression of nNOS in all groups (n = 5, *p<0.05).
(TIF)
