# Supplementary material for: β3-Adrenoreceptor Stimulation Protects against Myocardial Infarction Injury via eNOS and nNOS Activation
Source: PLoS One. 2014 Jun 9;9(6):e98713. doi: 10.1371/journal.pone.0098713 (PMC4049583; doi:10.1371/journal.pone.0098713)
Supplement: Methods S1 — RNA extraction and Reverse Transcription - Polymerase Chain Reaction (RT-PCR) analysis. Total RNA was extracted using TRIZOL reagent (Invitrogen) in accordance with the manufacturer's protocol. After reverse-transcribed, cDNA samples were subjected to polymerase chain reaction (PCR) amplification. The following primers were used: The primer sequence for nNOS: forward 5′-GGC ACT GGC ATC GCA CCC TT-3′, reverse 5′-CTT TGG CCT GTC CGG TTC CC-3′. The β-actin housekeeping gene was used as an endogenous internal control. The primers for β-actin: forward 5′-AAC CGC GAG AAG ATG ACC CAG ATC ATG TTT-3′; reverse, 5′-AGC AGC CGT GGC CAT CTC TTG CTC GAA GTC-3′. The PCR products were fractionated by 1.2% agarose gel electrophoresis and visualized under UV illumination after staining with ethidium bromide. (DOC) [file pone.0098713.s001.doc]

**Method S1**

**RNA extraction and Reverse Transcription - Polymerase Chain Reaction (RT-PCR) analysis**

Total RNA was extracted using TRIZOL reagent (Invitrogen) in accordance with the manufacturer’s protocol. After reverse-transcribed, cDNA samples were subjected to polymerase chain reaction (PCR) amplification. The following primers were used: The primer sequence for nNOS: forward 5’-GGC ACT GGC ATC GCA CCC TT-3’, reverse 5’-CTT TGG CCT GTC CGG TTC CC-3’. The β-actin housekeeping gene was used as an endogenous internal control. The primers for β-actin: forward 5’-AAC CGC GAG AAG ATG ACC CAG ATC ATG TTT-3’; reverse, 5’-AGC AGC CGT GGC CAT CTC TTG CTC GAA GTC-3’. The PCR products were fractionated by 1.2% agarose gel electrophoresis and visualized under UV illumination after staining with ethidium bromide.
