# Supplementary figures and images for: β3-Adrenoreceptor Stimulation Protects against Myocardial Infarction Injury via eNOS and nNOS Activation
Source: PLoS One. 2014 Jun 9;9(6):e98713. doi: 10.1371/journal.pone.0098713 (PMC4049583; doi:10.1371/journal.pone.0098713)

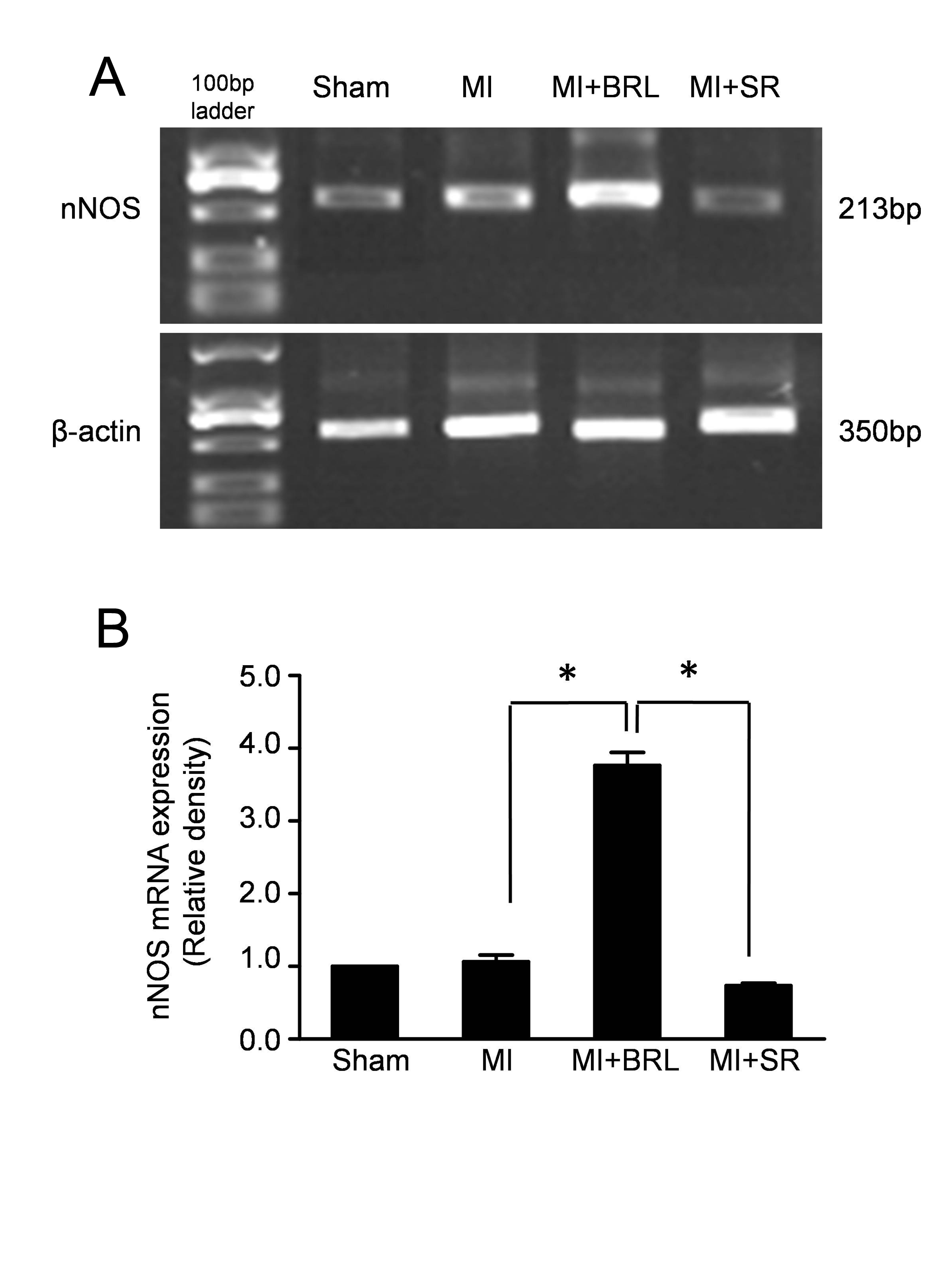

Supplement: Figure S1 — RT-PCR analysis of nNOS mRNA expression. A: PCR-based detection of nNOS in sham group,MI Group, MI+SR group and MI+BRL group. B: Semiquantitative analysis of the mRNA expression of nNOS in all groups (n = 5, *p<0.05). (TIF) [file pone.0098713.s002.tif]
